# Nomograms for the prediction of lateral lymph node metastasis in papillary thyroid carcinoma: Stratification by size

**DOI:** 10.3389/fonc.2022.944414

**Published:** 2022-09-28

**Authors:** Jia-Wei Feng, Jing Ye, Li-Zhao Hong, Jun Hu, Fei Wang, Sheng-Yong Liu, Yong Jiang, Zhen Qu

**Affiliations:** The Third Affiliated Hospital of Soochow University, Changzhou First People’s Hospital, Changzhou, China

**Keywords:** papillary thyroid carcinoma, papillary thyroid microcarcinoma, lateral lymph node metastasis, nomogram, surgery

## Abstract

**Background:**

Lateral lymph node metastasis (LLNM) is a risk factor of poor prognosis in papillary thyroid cancer (PTC). We aimed to determine predictive factors and develop the nomograms for LLNM in patients with papillary thyroid microcarcinoma (PTMC) and macro-PTC.

**Methods:**

We reviewed the medical records of 1,106 patients who underwent surgery between January 2019 and January 2022. Patients were divided into a PTMC and a macro-PTC group. We developed preoperative and postoperative nomograms for predicting LLNM based on results of multivariate analysis. Internal calibration was performed for these models.

**Results:**

The number of metastatic lymph nodes in lateral compartment was higher in macro-PTC patients. LLNM was independently associated with gender, the number of foci, location, shape, and central lymph node metastasis (CLNM) in PTMC patients. For macro-PTC patients, chronic lymphocytic thyroiditis, the number of foci, location, margin, CLNM, and central lymph node ratio were all independent predictors for LLNM. All the above factors were incorporated into nomograms, which showed the perfect discriminative ability.

**Conclusion:**

The diameter of the tumor has an impact on the rate of LLNM. Separate predictive systems should be used for PTMC and macro-PTC patients for more accurate clinical assessment of lateral lymph node status. Through these nomograms, we can not only detect high-risk patients with occult LLNM preoperatively, but also form appropriate treatment protocols for postoperative management of PTC patients with different risks.

## Introduction

The incidence of papillary thyroid cancer (PTC), which accounts for approximately 80.0% of thyroid cancers, has been increasing worldwide in recent decades (1). Although most patients with PTC have a good prognosis, the incidence of lymph node metastasis (LNM) is high, ranging from 20% to 90% (2–6). As reported, PTC patients with lateral lymph node metastasis (LLNM) had higher incidence of disease persistence, recurrence, and distant metastasis when compared to patients with or without central lymph node metastasis (CLNM) (7).

Unless suspicious LLNM is confirmed by preoperative fine needle aspiration cytology (FNAC), prophylactic lateral neck dissection (LND) is not recommended for patients with clinically negative (cN0) lateral neck (1). However, the incidence of occult LLNM was reported to be as high as 30.4% among PTC patients (8). Considering the presence of occult LLNM, which was hardly detected in the preoperative period, some patients who undergo thyroidectomy may detect the residual metastatic lymph nodes in the lateral compartment (9). Therefore, establishing predictive models for early detection of LLNM and residual risk is critical.

Tumor size is an important factor among the clinical and pathological features that can be assessed preoperatively and intraoperatively. With the increasing detection rate of papillary thyroid microcarcinoma (PTMC) (≤10 mm diameter) (1), the optimal treatment strategy of PTMC, especially whether PTMC needs surgery, remains controversial. Active surveillance has been recommended as a reasonable alternative approach to immediate surgery for low-risk PTMC according to a 10-year observational study (10). The possibility of LNM during active surveillance is a major concern for clinicians and patients. Clinically apparent lymph nodes re-stratified patients with PTMC from low risk to intermediate risk according to the 2015 American Thyroid Association (ATA) risk stratification (1). In addition, macro-PTC (>10 mm diameter) is more vulnerable to aggression. According to the previous research, tumor size was the best predictor of CLNM and LLNM and was significantly associated with lymph node recurrence (11). Therefore, we assumed that clinicopathological features may be different between PTMC and macro-PTC patients, and the clinical management of PTMC patients should be differentiated from macro-PTC patients.

Unlike previous studies that only determined risk factors of LLNM, we first aimed to investigate the differences in clinicopathological features between PTMC and macro-PTC patients, especially differences in LNM. Then, we aimed to perform subgroup analysis on this basis, investigating the risk factors for LLNM in PTMC and macro-PTC patients, respectively. Finally, we attempted to develop nomograms to predict LLNM. Through these accurate and easy-to-use nomograms, we can proactively detect high-risk patients with occult LLNM preoperatively and form appropriate treatment protocols for postoperative management of PTC patients with different risks.

## Materials and methods

### Study design

**Figure 1:**
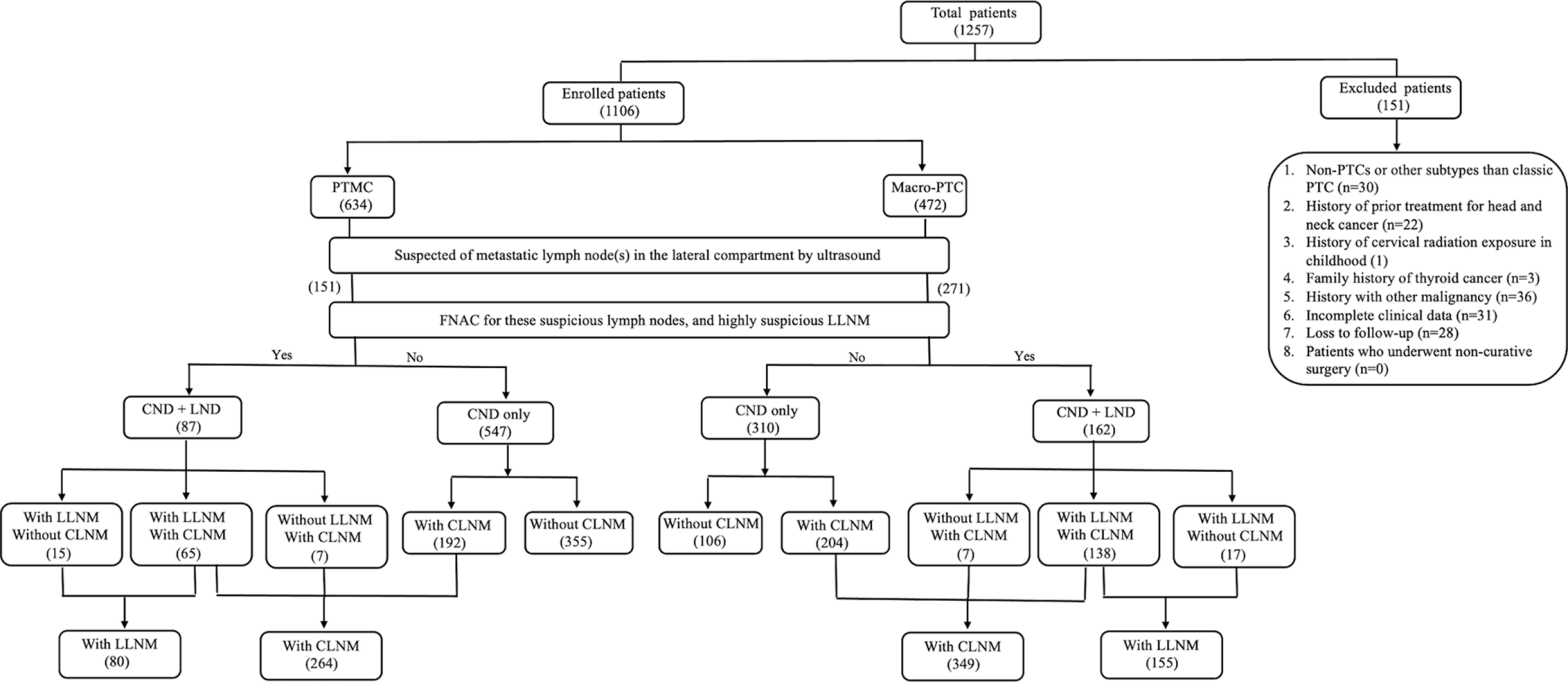
Flowchart of the patients enrolled in this study.

This retrospective study was approved by the Institutional Review Board of Changzhou First People’s Hospital, and the need for informed consent was waived due to the retrospective nature of this study. We retrospectively reviewed the medical records of 1,257 patients with pathologically proven PTC who underwent primary surgical treatment at our institution between January 2019 and January 2022. The following exclusion criteria were applied: (1) non-PTCs or other subtypes than classic PTC; (2) history of prior treatment for head and neck cancer; (3) history of cervical radiation exposure in childhood; (4) family history of thyroid cancer; (5) history with other malignancy; (6) incomplete clinical data; (7) loss to follow-up; and (8) patients who underwent non-curative surgery (residual tumor or lymph node detected within 6 months of initial surgery). A total of 1,106 patients were included (**Figure 1**).

### Preoperative examination and surgical procedures

Diagnostic medical sonographers with over 5 years of experience performed the high-resolution neck ultrasound to evaluate the lymph node status and thyroid nodules. Each thyroid nodule would be evaluated by the following features: shape, tumor site, nodular composition, echogenicity, margin, and echogenic foci. Cervical lymph nodes with the following characteristics were suspected of metastases: hyperechoic changes, roundness or necrosis, loss of the fatty hilum, microcalcification, or peripheral vascularity (12). FNAC was conducted to confirm the histopathologic diagnosis preoperatively for suspicious thyroid nodules and lateral lymph nodes.

Total thyroidectomy was performed if the patient had any of the following factors: tumor located in the thyroid isthmus, bilateral multifocality, tumor size >4.0 cm, or 1.0 cm < tumor size ≤ 4.0 cm with risk factors for recurrence, presence of extrathyroidal extension (ETE), and suspicious LLNM preoperatively (1). Otherwise, patients underwent lobectomy plus isthmectomy only. According to the Chinese guidelines for diagnosis and treatment of differentiated thyroids, central neck dissection (CND) was routinely performed for all PTC patients. On the premise of effectively protecting the parathyroid gland and recurrent laryngeal nerve (RLN), ipsilateral CND was performed for ipsilateral lesion; bilateral CND was performed for isthmus lesion and bilateral lesions. For patients with clinically suspicious unilateral LLNM confirmed by FNAC, total thyroidectomy plus CND and ipsilateral therapeutic lateral neck dissection (LND) was performed. CND included the removal of prelaryngeal, pretracheal, and paratracheal lymph nodes. LND referred to the removal of the lateral lymph nodes, including level II to V, while preserving the spinal accessory nerve, internal jugular vein, or sternocleidomastoid muscle. The status of cervical lymph nodes was confirmed by a final histological examination.

### Definitions

Body mass index (BMI) (kg/m^2^) was defined as weight (kg) divided by height (m) squared. According to the World Health Organization-BMI standard, enrolled PTC patients were divided into normal (BMI < 25 kg/m^2^), overweight (25 ≤ BMI < 30 kg/m^2^), and obese (BMI ≥ 30 kg/m^2^) group. The diagnosis of chronic lymphocytic thyroiditis (CLT) included any of the following: (i) elevated antibodies to thyroid peroxidase level (>50 IU/ml), and/or (ii) findings of diffuse heterogeneity on ultrasound, and/or (iii) diffuse lymphocytic thyroiditis on histopathology (13). ETE was defined as a tumor with capsular abutment of more than 25% of its perimeter on ultrasound (14). The tumor size and location were determined by the largest predominant primary lesion for multifocal lesions. For patients who did not undergo LND, the number of metastatic lymph nodes in lateral compartment was clinically considered to be zero. The central lymph node ratio (CLNR) was calculated as the ratio of metastatic lymph nodes in the central compartment out of the number of dissected lymph nodes in the central compartment.

### Postoperative complications

All patients underwent fiber laryngoscope before and after surgery to assess the mobility of vocal cords. Transient RLN injury was regarded as decreased or absence of vocal cord mobility resolving within 6 months of surgery. Impaired vocal cord mobility for more than 6 months after surgery was considered permanent RLN injury. Serum calcium and phosphorus concentrations were measured in all patients after surgery. Transient hypocalcemia was defined as an ionized calcium level <2.10 mmol/L during hospitalization and the calcium level returned to normal within 6 months. Permanent hypoparathyroidism was diagnosed in patients still requiring calcium supplementation more than 6 months after surgery.

### Statistical analysis

All statistical analysis was performed by using SPSS Version 25.0 software (Chicago, IL, USA), and R software Version 3.5.3 (The R Foundation for Statistical Computing). Pearson Chi-square test or Fisher’s exact test was used for categorical data, and independent *t*-test was used to compare continuous variables. Differences with *p* values less than 0.05 were regarded as significant. Binary logistic regression analysis was conducted to assess independent associations of LLNM with factors found to be statistically significant by univariate analysis. We constructed the risk prediction model Nomogram in R software according to independent factors screened through the logistic regression model. The discriminative power of the nomogram for predicting LLNM was determined using the area under the receiver operating characteristic (ROC) curve, also known as the concordance index, with values ranging from 0.50 to 1.00. To address model overfitting and obtain a relatively unbiased evaluation, we used 1,000 random bootstrap resamples. The calibration of diagnostic nomogram was further evaluated by the calibration chart, which plotted the predicted probability of the nomogram against the observed probability.

## Results

### Baseline clinicopathological characteristics of PTC patients with different sizes

In our study, 151 PTMC patients and 271 macro-PTC patients were suspected of LLNM by preoperative ultrasound and underwent FNAC for these suspicious lymph nodes. Eighty-seven PTMC patients and 162 macro-PTC patients had high suspicion of LLNM and underwent LND. Postoperative pathology revealed LLNM in 80 PTMC patients and 155 macro-PTC patients (**Figure 1**).

**Table 1** shows the clinicopathological characteristics of the enrolled 1,106 PTC patients in this study. The 1,106 patients consisted of 800 women (72.3%) and 306 men (27.7%). There were 196 patients (17.7%) aged 55 years or older. CLT was present in 370 patients (33.5%) and absent in the remaining 736 patients (66.5%). The number of patients with one, two, and more than two foci in the thyroid gland were 712 (64.4%), 268 (24.2%), and 126 (11.4%), respectively. Tumors located in the upper portion of the thyroid gland were detected in 553 patients (50.0%), and tumors located in the middle/lower lobe of thyroid were detected in 553 patients (50.0%). In this study, tumors located in the isthmus were included in the group with tumors located in the middle pole. There were 74 cases of isthmus tumors, including 44 cases in the PTMC group and 30 cases in the macro-PTC group.

**Table 1:** Clinicopathological features of PTC patients with different tumor sizes.

Characteristics	Total	PTMC	Macro-PTC	*p-*value
	1,106	634	472	
Sex				
Male	306 (27.7%)	150 (23.7%)	156 (33.1%)	
Female	800 (72.3%)	484 (76.3%)	316 (66.9%)	0.001
Age (years)				
≥55	196 (17.7%)	113 (17.8%)	83 (17.6%)	
<55	910 (82.3%)	521 (82.2%)	389 (82.4%)	0.918
BMI (kg/m^2^)				
Normal	123 (11.1%)	80 (12.6%)	43 (9.1%)	
Overweight	592 (53.5%)	350 (55.2%)	242 (51.3%)	
Obesity	391 (35.4%)	204 (32.2%)	187 (39.6%)	0.018
Diabetes				
Absence	964 (87.2%)	542 (85.5%)	422 (89.4%)	
Presence	142 (12.8%)	92 (14.5%)	50 (10.6%)	0.054
CLT				
Absence	736 (66.5%)	411 (64.8%)	325 (68.9%)	
Presence	370 (33.5%)	223 (35.2%)	147 (31.1%)	0.160
BRAF V600E mutation				
Negative	142 (12.8%)	73 (11.5%)	69 (14.6%)	
Positive	964 (87.2%)	561 (88.5%)	403 (85.4%)	0.127
The number of foci				
1	712 (64.4%)	435 (68.6%)	277 (58.7%)	
2	268 (24.2%)	144 (22.7%)	124 (26.3%)	
3 or more	126 (11.4%)	55 (8.7%)	71 (15.0%)	<0.001
Multifocality				
Solitary	712 (64.4%)	435 (68.6%)	277 (58.7%)	
Ipsilateral multifocality	150 (13.6%)	87 (13.7%)	63 (13.3%)	
Bilateral multifocality	244 (22.1%)	112 (17.7%)	132 (28.0%)	<0.001
Location				
Upper	553 (50.0%)	301 (47.5%)	252 (53.4%)	
Middle/Lower Upper	553 (50.0%)	333 (52.5%)	220 (46.6%)	0.052
Nodular composition				
Mixed cystic and solid	10 (0.9%)	4 (0.6%)	6 (1.3%)	
Solid	1,096 (99.1%)	630 (99.4%)	466 (98.7%)	0.269
Echogenicity				
Hyperechoic or isoechoic	33 (3.0%)	12 (1.9%)	21 (4.4%)	
Hypoechoic	1,057 (95.6%)	616 (97.2%)	441 (93.4%)	
Very hypoechoic	16 (1.4%)	6 (0.9%)	10 (2.1%)	0.012
Shape				
A/T ≤1	710 (64.2%)	455 (71.8%)	255 (54.0%)	
A/T >1	396 (35.8%)	179 (28.2%)	217 (46.0%)	<0.001
Margin				
Smooth	646 (58.4%)	435 (68.6%)	211 (44.7%)	
Lobulated or irregular	289 (26.1%)	131 (20.7%)	158 (33.5%)	
ETE	171 (15.5%)	68 (10.7%)	103 (21.8%)	<0.001
Echogenic foci				
None or large comet-tail artifacts	319 (28.8%)	230 (36.3%)	89 (18.9%)	
Macrocalcifications	67 (6.1%)	36 (5.7%)	31 (6.6%)	
Peripheral calcifications	8 (0.7%)	0 (0.0%)	8 (1.7%)	
Punctate echogenic foci	712 (64.4%)	368 (58.0%)	344 (72.9%)	<0.001
CLNM				
Absence	493 (44.6%)	370 (58.4%)	123 (26.1%)	
Presence	613 (55.4%)	264 (41.6%)	349 (73.9%)	<0.001
LLNM				
Absence	871 (78.8%)	554 (87.4%)	317 (67.2%)	
Presence	235 (21.2%)	80 (12.6%)	155 (32.8%)	<0.001
No. of removed LNs in CC	7.7 ± 4.9 (2–35)	7.5 ± 4.6 (2–25)	8.1 ± 5.1 (2–35)	0.031
No. of metastatic LNs in CC	2.0 ± 2.9 (0–18)	1.2 ± 2.2 (0–15)	3.1 ± 3.3 (0–18)	<0.001
No. of removed LNs in LC*	28.0 ± 10.3 (10–69)	28.1 ± 10.7 (11–57)	27.9 ± 10.2 (10–69)	0.881
No. of metastatic LNs in LC*	5.2 ± 4.1 (0–22)	4.3 ± 3.1 (0–13)	5.6 ± 4.5 (0–22)	0.006

PTC, papillary thyroid carcinoma; PTMC, papillary thyroid microcarcinoma; BMI, body mass index; CLT, chronic lymphocytic thyroiditis; A/T, aspect ratio (height divided by width on transverse views); ETE, extrathyroidal extension; LN, lymph node; CLNM, central lymph node metastasis; LLNM, lateral lymph node metastasis; CC, central compartment; LC, lateral compartment.

The categorical variables were expressed as n (%).

The continuous variables were expressed as the mean ± standard deviations (range).

*The number of metastatic and removed lymph nodes in lateral compartment was clinically considered to be zero for patients who did not undergo lateral neck dissection.

**Figure 2:**
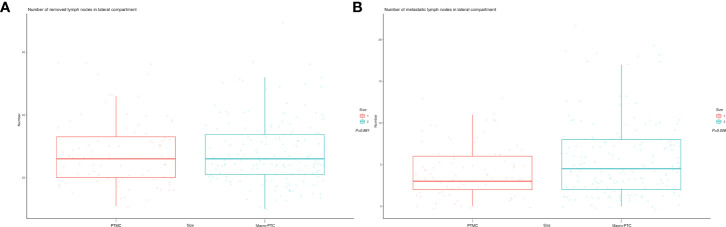
Boxplot of number of lymph nodes. **(A)** Number of removed lymph nodes in lateral compartment: PTMC group vs. macro-PTC group: 28.1 ± 10.7 vs. 27.9 ± 10.2, *p* = 0.881; **(B)** number of metastatic lymph nodes in lateral compartment: PTMC group vs. macro-PTC group: 4.3 ± 3.1 vs. 5.6 ± 4.5, *p* = 0.006.

We divided patients into the PTMC group and the macro-PTC group according to the largest diameter of tumor. There were 634 patients in the PTMC group and 472 patients in the macro-PTC group. There was a statistically significant difference between two groups in terms of sex, BMI, number of foci, echogenicity, shape, margin, echogenic foci, CLNM, and LLNM (all *p* < 0.05). The number of removed lymph nodes in the central compartment of macro-PTC patients was more than PTMC patients (8.1 ± 5.1 vs. 7.5 ± 4.6, *p* = 0.031). However, there was no statistical difference in the number of removed lymph nodes in the lateral compartment between the two groups (*p* = 0.881). The number of metastatic lymph nodes in the central compartment and lateral compartment was fewer in the PTMC group when compared with the macro-PTC group (1.2 ± 2.2 vs. 3.1 ± 3.3; 4.3 ± 3.1 vs. 5.6 ± 4.5, respectively, all *p* < 0.05). **Figure 2** showed the boxplot of the number of removed and metastatic lymph nodes in the lateral compartment. As for other clinicopathological factors, no significant differences were observed.

Of 634 PTMC patients, 12 patients developed RLN injury (including 7 patients with transient RLN injury and 5 patients with permanent RLN injury). As for 472 macro-PTC patients, 7 patients developed RLN injury (including 4 patients with transient RLN injury and 3 patients with permanent RLN injury). Hypocalcemia occurred in 25 PTMC patients, of whom 19 cases were temporary and 6 cases were permanent. As for macro-PTC patients, 11 cases developed hypocalcemia, including 7 temporary cases and 4 permanent cases. There was no statistical difference in the incidence of RLN injury and hypocalcemia between PTMC and macro-PTC patients.

### Prevalence and distribution of metastatic lymph nodes in the lateral neck

As shown in **Figure 1**, among 634 PTMC patients, 87 patients had suspected metastatic lymph nodes in the lateral compartment, and of 472 macro-PTC patients, 162 patients had suspected metastatic lymph nodes in the lateral compartment. A total of 249 PTC patients underwent the total thyroidectomy plus CND and ipsilateral therapeutic LND. Postoperative pathology showed that 80 PTMC patients developed LLNM, and 155 macro-PTC patients developed LLNM.

**Table 2** shows the distribution of metastatic lymph nodes in the lateral compartment of PTMC and macro-PTC patients. Simultaneous lymph node metastasis in level II, III, and IV was the most common in PTMC and macro-PTC patients, which is up to 21.8% and 24.7%, respectively. For the rate of metastatic lymph nodes in the lateral compartment divided by region, level IV metastasis was the most common in PTMC and macro-PTC patients (75.9% for PTMC, 79.6% for macro-PTC), followed by level III metastasis (69.0% for PTMC, 73.5% for macro-PTC); level V metastasis was the least common (20.7% for PTMC, 23.5% for macro-PTC).

**Table 2:** Distribution of metastatic LNs in the LC of 249 PTC patients who underwent LND.

Distribution of LLNM	PTMC87	Macro-PTC162	*p*-value
Without LLNM	7 (8.0%)	7 (4.3%)	0.353
Single level			
II	1 (1.1%)	0 (0.0%)	0.146
III	7 (8.0%)	15 (9.3%)	0.748
IV	11 (12.6%)	21 (13.0%)	0.943
V	0 (0.0%)	1 (0.6%)	0.353
Two levels			
II+III	3 (3.4%)	5 (3.1%)	0.878
II+IV	6 (6.9%)	5 (3.1%)	0.284
II+V	0 (0.0%)	1 (0.6%)	0.353
III+IV	15 (17.2%)	31 (19.1%)	0.713
III+V	0 (0.0%)	3 (1.9%)	0.504
IV+V	2 (2.3%)	7 (4.3%)	0.646
Three levels			
II+III+IV	19 (21.8%)	40 (24.7%)	0.614
II+III+V	3 (3.4%)	1 (0.6%)	0.099
II+IV+V	0 (0.0%)	1 (0.6%)	0.353
III+IV+V	9 (10.3%)	7 (4.3%)	0.065
Four levels			
II+III+IV+V	4 (4.6%)	17 (10.5%)	0.110
Summary of LLNM			
II	36 (41.4%)	70 (43.2%)	0.781
III	60 (69.0%)	119 (73.5%)	0.452
IV	66 (75.9%)	129 (79.6%)	0.492
V	18 (20.7%)	38 (23.5%)	0.618

LN, lymph node; LC, lateral compartment; LLNM, lateral lymph node metastasis; PTC, papillary thyroid carcinoma; PTMC, papillary thyroid microcarcinoma.

### Risk factors for LLNM in PTMC patients

We first analyzed the risk factors for LLNM in PTMC patients. As shown in **Table 3**, LLNM presented the significant association with sex, tumor size, the number of foci, location, shape, echogenic foci, CLNM, and CLNR in the univariate analysis (all *p* < 0.05).

**Table 3:** Univariate analysis and multivariate analysis of factors associated with LLNM in patients with PTMC.

Characteristics	LLNM					Multivariate analysis							
	Presence (*n* = 80)		Absence (*n* = 554)	*p*-value		Adjusted OR (95% CI)		*p*-value		Score^1^		Score^2^	
Sex													
Female	33 (41.3%)		117 (21.1%)			Ref				0		0	
Male	47 (58.8%)		437 (78.9%)	<0.001		2.011 (1.162–3.482)		0.013		60		35	
Age (years)													
≥55	11 (13.8%)		102 (18.4%)										
<55	69 (86.3%)		452 (81.6%)	0.308									
BMI (kg/m^2^)													
Normal	4 (5.0%)		76 (13.7%)										
Overweight	50 (62.5%)		300 (54.2%)										
Obesity	26 (32.5%)		178 (32.1%)	0.078									
Diabetes													
Absence	72 (90.0%)		470 (84.8%)										
Presence	8 (10.0%)		84 (15.2%)	0.220									
CLT													
Absence	56 (70.0%)		355 (64.1%)										
Presence	24 (30.0%)		199 (35.9%)	0.300									
BRAF V600E mutation													
Negative	6 (7.5%)		67 (12.1%)										
Positive	74 (92.5%)		487 (87.9%)	0.229									
Maximum tumor size													
≤0.5 cm	7 (8.8%)		119 (21.5%)			Ref							
>0.5 to ≤1 cm	73 (91.3%)		435 (78.5%)	0.008		1.394 (0.575–3.375)		0.462					
The number of foci													
1	41 (51.2%)		394 (71.1%)			Ref				0		0	
2	23 (28.7%)		121 (21.8%)			2.050 (1.106–3.801)		0.023		56		36	
3 or more	16 (20.0%)		39 (7.0%)	<0.001		3.581 (1.667–7.693)		0.001		100		64	
Bilateral tumors													
Absence	64 (80.0%)		458 (82.7%)										
Presence	16 (20.0%)		96 (17.3%)	0.558									
Location													
Middle/Lower	27 (33.8%)		306 (55.2%)			ref				0		0	
Upper	53 (66.3%)		248 (44.8%)	<0.001		2.623 (1.519–4.530)		0.001		61		48	
Nodular composition													
Mixed cystic and solid	0 (0.0%)		4 (0.7%)										
Solid	80 (100.0%)		550 (99.3%)	0.298									
Echogenicity													
Hyperechoic or isoechoic	0 (0.0%)		12 (2.2%)										
Hypoechoic	78 (97.5%)		538 (97.1%)										
Very hypoechoic	2 (2.5%)		4 (0.7%)	0.084									
Shape													
A/T ≤1	42 (52.5%)		413 (74.5%)			Ref				0		0	
A/T >1	38 (47.5%)		141 (25.5%)	<0.001		2.455 (1.424–4.235)		0.001		69		45	
Margin													
Smooth	52 (65.0%)		383 (69.1%)										
Lobulated or irregular	21 (26.3%)		110 (19.9%)										
ETE	7 (8.8%)		61 (11.0%)	0.389									
Echogenic foci													
None/large comet-tail artifacts		15 (18.8%)	215 (38.8%)				Ref						
Macrocalcifications	10 (12.5%)		26 (4.7%)				2.378 (0.764–7.403)	0.135					
Peripheral calcifications	0 (0.0%)		0 (0.0%)				–	–					
Punctate echogenic foci	55 (68.8%)		313 (56.5%)		<0.001		1.349 (0.691–2.634)	0.381					
CLNM													
Absence	13 (16.3%)		357 (64.4%)			Ref						0	
Presence	67 (83.8%)		197 (35.6%)	<0.001		7.390 (3.903–13.990)		<0.001				100	
CLNR													
<0.5	46 (57.5%)		483 (87.2%)			Ref							
≥0.5	34 (42.5%)		71 (12.8%)	<0.001		1.586 (0.869–2.896)		0.133					
Largest size of lymph node in LC													
≤2 cm	63 (78.8%)		453 (81.8%)										
>2 cm	17 (21.3%)		101 (18.2%)	0.517									

LLNM, lateral lymph node metastasis; PTMC, papillary thyroid microcarcinoma; BMI, body mass index; CLT, chronic lymphocytic thyroiditis; A/T, aspect ratio (height divided by width on transverse views); ETE, extrathyroidal extension; CLNM, central lymph node metastasis; CLNR, central lymph node ratio; LC, lateral compartment; OR, odds ratio; CI, confidence interval.

The categorical variables were expressed as n (%).

Score^1^ represents the preoperative score of the preoperative model. Score^2^ represents the postoperative score of the postoperative model.

Multivariate logistic regression modeling was further conducted to screen for significant variables associated with LLNM in PTMC patients. Multivariate analysis showed that sex (OR: 2.011, 95% CI: 1.162–3.482, *p* = 0.013), two tumor foci (OR: 2.050, 95% CI: 1.106–3.801, *p* = 0.023), three or more tumor foci (OR: 3.581, 95% CI: 1.667–7.693, *p* = 0.001), tumor located in the upper pole (OR: 2.623, 95% CI: 1.519–4.530, *p* = 0.001), tumor with aspect ratio (A/T) >1 (OR: 2.455, 95% CI: 1.424–4.235, *p* = 0.001), and presence of CLNM (OR: 7.390, 95% CI: 3.903–13.990, *p* < 0.001) remained independent predictors for LLNM in PTMC patients.

### Risk factors for LLNM in macro-PTC patients

The relationships between predictive factors and LLNM in macro-PTC patients are presented in **Table 4**. Sex, BMI, CLT, tumor size, the number of foci, location, margin, CLNM, and CLNR were all correlated with LLNM by univariate analysis (all *p* < 0.05).

**Table 4:** Univariate analysis and multivariate analysis of factors associated with LLNM in patients with macro-PTC.

Characteristics	LLNM				Multivariate analysis				
	Presence (*n* = 155)	Absence (*n* = 317)		*p*-value	Adjusted OR (95% CI)	*p*-value	Score^1^	Score^2^	
Sex									
Female	91 (58.7%)	225 (71.0%)			Ref				
Male	64 (41.3%)	92 (29.0%)		0.008	1.046 (0.646–1.692)	0.855			
Age (years)									
≥55	25 (16.1%)	58 (18.3%)							
<55	130 (83.9%)	259 (81.7%)		0.561					
BMI (kg/m^2^)									
Normal	6 (3.9%)	37 (11.7%)			Ref				
Overweight	81 (52.3%)	161 (50.8%)			1.902 (0.703–5.147)	0.205			
Obesity	68 (43.9%)	119 (37.5%)		0.018	2.465 (0.902–6.738)	0.079			
Diabetes									
Absence	138 (89.0%)	284 (89.6%)							
Presence	17 (11.0%)	33 (10.4%)		0.853					
CLT									
Presence	31 (20.0%)	116 (36.6%)			Ref		0	0	
Absence	124 (80.0%)	201 (63.4%)		<0.001	1.807 (1.080–3.024)	0.024	48	45	
BRAF V600E mutation									
Negative	23 (14.8%)	46 (14.5%)							
Positive	132 (85.2%)	271 (85.5%)		0.925					
Maximum tumor size									
>1 to ≤2 cm	77 (49.7%)	199 (62.8%)			Ref				
>2 to ≤4 cm	67 (43.2%)	98 (30.9%)			1.402 (0.871–2.256)	0.165			
≥4 cm	11 (7.1%)	20 (6.3%)		0.022	1.284 (0.514–3.206)	0.593			
The number of foci									
1	76 (49.0%)	201 (63.4%)			Ref		0	0	
2	41 (26.5%)	83 (26.2%)			1.027 (0.614–1.720)	0.918	7	2	
3 or more	38 (24.5%)	33 (10.4%)		<0.001	2.588 (1.383–4.841)	0.003	70	73	
Bilateral tumors									
Absence	107 (69.0%)	233 (73.5%)							
Presence	48 (31.0%)	84 (26.5%)		0.310					
Location									
Middle/Lower	49 (31.6%)	171 (53.9%)			Ref		0	0	
Upper	106 (68.4%)	146 (46.1%)		<0.001	2.139 (1.360–3.365)	0.001	56	58	
Nodular composition									
Mixed cystic and solid	2 (1.3%)	4 (1.3%)							
Solid	153 (98.7%)	313 (98.7%)		0.979					
Echogenicity									
Hyperechoic or isoechoic	4 (2.6%)	17 (5.4%)							
Hypoechoic	147 (94.8%)	294 (92.7%)							
Very hypoechoic	4 (2.6%)	6 (1.9%)		0.322					
Shape									
A/T ≤1	90 (58.1%)	165 (52.1%)							
A/T >1	65 (41.9%)	152 (47.9%)		0.218					
Margin									
Smooth	38 (24.5%)	173 (54.6%)			Ref		0	0	
Lobulated or irregular	63 (40.6%)	95 (30.0%)			2.378 (1.410–4.009)	0.001	76	66	
ETE	54 (34.8%)	49 (15.5%)		<0.001	3.691 (2.081–6.549)	<0.001	100	100	
Echogenic foci									
None/large comet-tail artifacts	21 (13.5%)	68 (21.5%)						
Macrocalcifications	14 (9.0%)	17 (5.4%)							
Peripheral calcifications	2 (1.3%)	6 (1.9%)							
Punctate echogenic foci	118 (76.1%)	226 (71.3%)		0.100					
CLNM									
Absence	13 (8.4%)	110 (34.7%)			Ref			0	
Presence	142 (91.6%)	207 (65.3%)		<0.001	2.621 (1.273–5.398)	0.009		74	
CLNR									
<0.5	52 (33.5%)	219 (69.1%)			Ref			0	
≥0.5	103 (66.5%)	98 (30.9%)		<0.001	2.359 (1.439–3.866)	0.001		66	
Largest size of lymph node in LC									
≤2 cm	127 (81.9%)	270 (85.2%)							
>2 cm	28 (18.1%)	47 (14.8%)		0.366					

LLNM, lateral lymph node metastasis; PTC, papillary thyroid carcinoma; BMI, body mass index; CLT, chronic lymphocytic thyroiditis; A/T, aspect ratio (height divided by width on transverse views); ETE, extrathyroidal extension; CLNM, central lymph node metastasis; CLNR, central lymph node ratio; LC, lateral compartment; OR, odds ratio; CI, confidence interval.

The categorical variables were expressed as n (%).

Score^1^ represents the preoperative score of the preoperative model.

Score^2^ represents the postoperative score of the postoperative model.

Multivariate analysis was performed to determine whether these parameters were independently correlated LLNM in macro-PTC. Absence of CLT (OR: 1.807, 95% CI: 1.080–3.024, *p* = 0.024), three or more tumor foci (OR: 2.588, 95% CI: 1.383–4.841, *p* = 0.003), tumor located in the upper pole (OR: 2.139, 95% CI: 1.360–3.365, *p* = 0.001), lobulated or irregular tumor (OR: 2.378, 95% CI: 1.410–4.009, *p* = 0.001), presence of ETE (OR: 3.691, 95% CI: 2.081–6.549, *p* < 0.001), presence of CLNM (OR: 2.621, 95% CI: 1.273–5.398, *p* = 0.009), and CLNR (OR: 2.359, 95% CI: 1.439–3.866, *p* = 0.001) remained independently predictive of LLNM in macro-PTC patients.

### Development of nomograms for predicting LLNM in PTMC and macro-PTC patients

**Figure 3:**
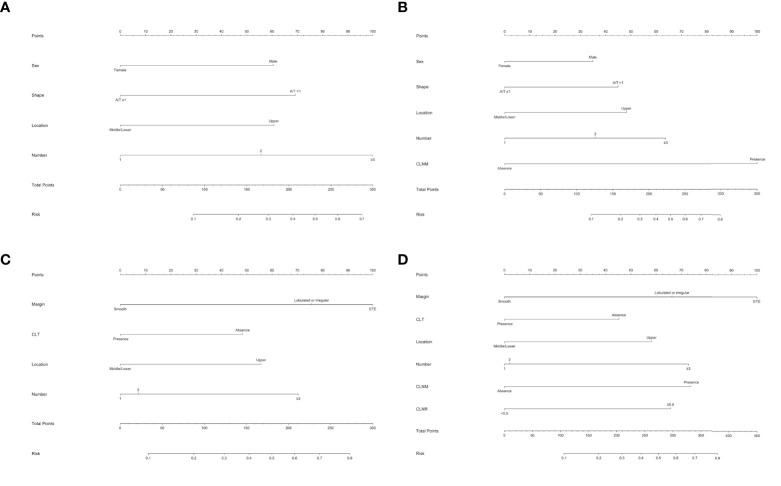
Nomogram for predicting LNM in patients with PTC. **(A)** Preoperative nomogram for predicting LLNM in PTMC patients; **(B)** postoperative nomogram for predicting LLNM in PTMC patients; **(C)** preoperative nomogram for predicting LLNM in macro-PTC patients; **(D)** postoperative nomogram for predicting LLNM in macro-PTC patients.

To better predict the individual probability of LLNM, we constructed a series of diagnostic nomograms using independent predictors selected by binary logistic regression analysis to generate a combined measurement (**Figure 3**). Because none of the leading guidelines recommend prophylactic LND, we divided models of LLNM into the preoperative model and the postoperative model for PTMC and macro-PTC patients. Preoperative models (**Figures 3A, C**) were constructed based on clinical factors, and postoperative models (**Figures 3B, D**) were constructed based on clinicopathological factors. Detailed factors are listed in the **Tables 3** and **4**. According to the regression coefficient of LLNM, each variable was proportionally distributed as the point in the range of 0 to 100 in the nomograms. Detailed scores are listed in the **Tables 3** and **4**. The corresponding probability of LLNM in each person can be determined by adding the total score and positioning it on the scale of the total score.

### Internal validation of the prediction nomograms

**Figure 4:**
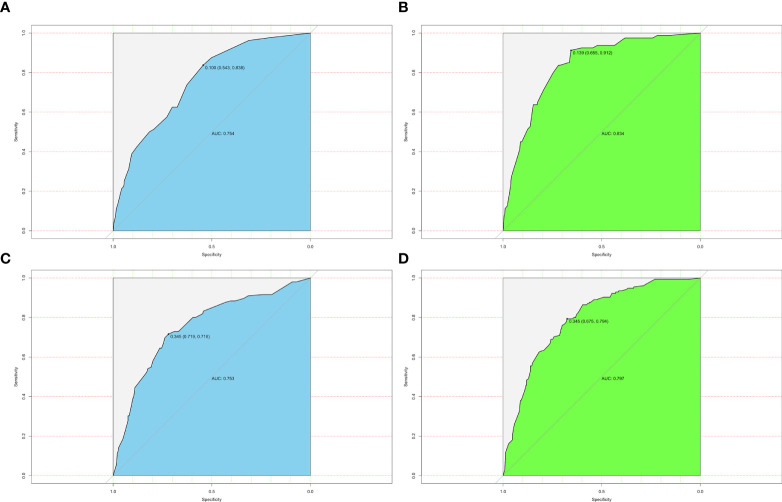
ROC curves for different models. **(A)** AUC was 0.754 for the preoperative model of predicting LLNM in PTMC patients; **(B)** AUC was 0.834 for the postoperative model of predicting LLNM in PTMC patients; **(C)** AUC was 0.753 for the preoperative model of predicting LLNM in macro-PTC patients; **(D)** AUC was 0.797 for the postoperative model of predicting LLNM in macro-PTC patients.

The ROC analysis for nomograms of LLNM was then performed. Area under curves (AUCs) for the preoperative model and postoperative model for predicting LLNM in PTMC patients were 0.754 and 0.834, respectively (**Figures 4A, B**). Moreover, for predicting LLNM in macro-PTC patients, AUCs for the preoperative model and postoperative model were 0.753 and 0.797, respectively (**Figures 4C, D**).

**Figure 5:**
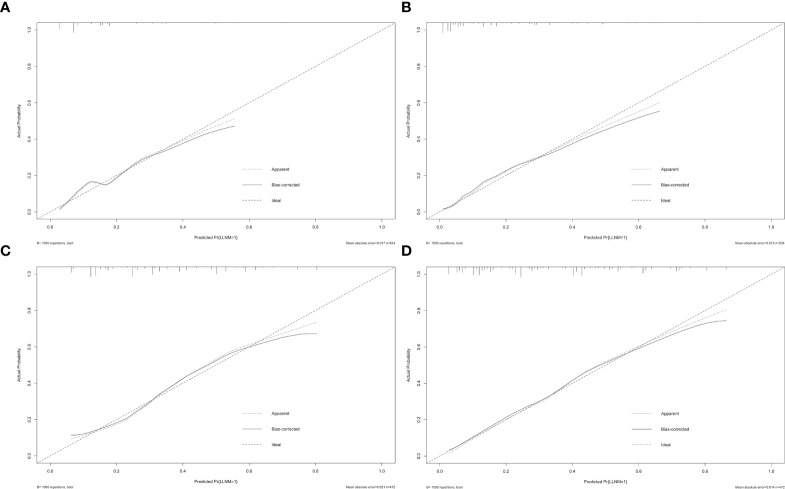
Calibration curves of nomograms for predicting LLNM. The diagonal dashed line represents the ideal prediction by the perfect nomogram; the solid line represents the calibration estimate from the internally validated model; the dotted line indicates the apparent predictive accuracy. The closer the solid line is to the dotted line, the stronger the predictive ability of the model. **(A)** Calibration curve for the preoperative model of predicting LLNM in PTMC patients; **(B)** calibration curve for the postoperative model of predicting LLNM in PTMC patients; **(C)** calibration curve for the preoperative model of predicting LLNM in macro-PTC patients; **(D)** calibration curve for the postoperative model of predicting LLNM in macro-PTC patients.

Furthermore, the similar bootstrap resampling procedure was used to conduct the internal calibration plot for established models. The calibration curve of nomograms presented good agreement between the predicted and observed probability of LLNM. After the adjustment for optimism, corrected risks also showed excellent agreement with observed metastasis risk, and only minor discrepancies were observed (**Figure 5**).

Moreover, we developed the nomogram for LLNM in all PTC patients and compared this nomogram with nomograms for LLNM in PTMC and macro-PTC, respectively (**Supplementary Figures 1**–**3**). We found that the AUCs of nomograms for LLNM in PTMC and macro-PTC were higher than that in all PTC patients. It shows that we are correct to develop the nomogram of LLNM separately for PTMC and macro-PTC.

## Discussion

LLNM, which is defined as level II–V cervical lymph node involvement, suggests a poor prognosis among PTC patients (7). LND is only recommended for PTC patients with positive FNAC-proven LLNM according to the ATA guidelines (1). Notably, ultrasound-guided FNAC is not available in some institutions, and the false-negative rate of FNAC can be as high as 16.7% (15). Accurate evaluation factor for LLNM is necessary for distinguishing high-risk PTC patients.

LNM usually occurs in a stepwise and continuous manner. LLNM usually occurs after CLNM, which explains the higher incidence of CLNM than LLNM. However, there are some special cases. For example, 15 (2.4%) and 17 (3.6%) patients in the PTMC and macro-PTC groups developed skip metastasis (negative CLNM with positive LLNM). Patients often had multi-level metastasis, and simultaneous metastasis in levels II, III, and IV was the most common in both PTMC and macro-PTC. Within the lateral cervical lymph node chain, level IV metastasis was consistently the most common in both PTMC and macro-PTC, followed by level III, level II, and level V. These findings were consistent with a previous study (16).

In our study, we found that male gender, multifocality, upper location of tumor, tumor with A/T >1, and presence of CLNM were risk factors for LLNM in PTMC patients. These findings were consistent with published articles. Consistent with a previous study including 1,066 patients with PTMC, our results showed that an increase in the number of tumors led to an increased risk of LLNM (17). Multifocality leading to increased aggressiveness may be due to the fact that multifocal clonal origins result from intraglandular spread of a single primary tumor (18). Liu et al. (19) also found upper portion location was the risk factor for LLNM in PTMC. The lymphatic drainage system of the upper pole differs from that of other parts of the thyroid lobe. Tumors in the upper lobe can spread directly to the ipsilateral lateral chamber through the lymphatic vessels along the superior venous vessels, which lead to the higher rate of LLNM in upper pole location. In addition, in rare cases, some tumors can even bypass the central compartment and directly metastasize to the lateral compartment through these channels, which is known as skip metastasis (20, 21). The diagnosis of PTMC has also improved with the development of ultrasound. However, studies linking LNM to ultrasound features of PTC are limited. We found a significant difference in the probability of developing LLNM in PTMC with A/T >1. Other ultrasound features, such as nodular composition, echogenicity, and echogenic foci, were not associated with LLNM in PTMC patients. In several studies, CLNM was shown to be an important factor for LLNM in PTMC (22, 23), and our study also confirmed this correlation.

Then, we compared the clinicopathological characteristics predictive of LLNM in patients with macro-PTC. Multivariate analyses indicated that absence of CLT, three or more tumor foci, upper location of tumor, lobulated or irregular tumor, presence of ETE, presence of CLNM, and CLNR were all independent predictors for LLNM in macro-PTC patients. These were consistent with the known high-risk features of macro-PTC. In macro-PTC, risk factors for LLNM such as multifocality, location, and CLNM are also risk factors for LLNM in PTMC. CLT has been considered a risk factor for the development of thyroid malignancy. However, data on the effect of CLT on cervical LNM in PTC were inconsistent. Some studies showed that PTC patients coexistent with CLT had a higher incidence of LNM (24), while others showed the opposite conclusion (25, 26). In our study, the most significant result is the association of CLT with the less frequent LLNM. This result is in agreement with the meta-analysis of Lee et al. (27), that the lymphocytic infiltration counteracts tumor progression. Therefore, we infer that concurrent CLT is a protective factor for macro-PTC patients. As for macro-PTC, ultrasound features, such as lobulated or irregular tumor and presence of ETE, were associated with LLNM. Ultrasound showed high sensitivity (80%) for predicting minimal ETE in PTC patients (28). In this study, a tumor was classified as suspicious for ETE when there is a contact of >25% with the adjacent capsule of PTC. Metastatic ratio could be used to quantitatively evaluate the positive central lymph nodes. We analyzed the metastatic ratio of central lymph nodes and set the cutoff metastatic ratio as 50% according to previous studies (29, 30). We found that CLNR was significantly associated with LLNM in macro-PTC.

Considering none of the leading guidelines to date recommend prophylactic LND, we incorporated all the above factors into nomograms to create possibilities for detecting high-risk patients with occult LLNM preoperatively and providing an individualized plan for postoperative management of PTC patients. Although there was no statistical difference in the incidence of RLN injury and hypocalcemia in PTMC and macro-PTC patients, the incidence of CLNM and LLNM in macro-PTC was much higher than that of PTMC (73.9% vs. 41.6%; 32.8% vs. 12.6%, respectively), indicating that the diameter of the tumor has an impact on the rate of LNM, and separate predictive systems should be used for PTMC and macro-PTC patients for more accurate clinical assessment of lateral lymph node status. We separately established two predictive nomograms to predict LLNM in PTMC patients before and after operation. In the same way, we built predictive nomograms to predict LLNM in macro-PTC patients. All the above nomograms showed excellent precision. In addition, compared with the nomogram for LLNM in all PTC patients, nomograms for LLNM in PTMC and macro-PTC all showed higher AUCs. The largest contributors to nomogram scores differed between PTMC and macro-PTC. The number of foci and CLNM were the largest contributors for the preoperative and postoperative model in PTMC, respectively. As for macro-PTC, the margin was the largest contributor to both preoperative and postoperative models. These findings suggest that preoperative attention should be paid to the number of tumors for PTMC patients, and the preoperative tumor margin status is extremely important for macro-PTC. Experienced sonographers should perform detailed preoperative examinations to detect more suspected lesions in PTMC patients and accurately assess the margin of macro-PTC. Moreover, for candidates of active surveillance of PTMC, we can provide these patients more information to help them decide whether to participate in active surveillance based on the preoperative nomogram. Combined with other risk factors, high-resolution ultrasound by experienced sonographers should be performed to detect small metastatic lymph nodes in the lateral compartment early for patients with a high risk of LLNM according to preoperative nomograms. Experienced surgeons are recommended to perform detailed operations on these patients. Moreover, considering the possibility of performing LND in the future, carbon nanoparticle suspension injection should be used at the first surgery to prevent missing small metastatic lymph nodes. In addition to assisting in preoperative screening high-risk patients of LLNM, our postoperative nomograms may be helpful in detecting the risk of residual LLNM postoperatively for PTC patients who did not undergo LND. For patients with a high risk of LLNM, we can increase the frequency of follow-up and ultrasound, and decrease the cutoff of FNAC for suspicious lymph nodes in the lateral compartment. Adjuvant radioactive iodine should be carried out to detect and address possible residual carcinoma in the lateral compartment when necessary. Unless obvious clinical evidence of LLNM is present, “wait and see” is recommended for patients with a low risk of LLNM.

Despite the fact that some encouraging results were achieved, this study still had some limitations. First, although our study has a large sample size, it is a retrospective study, which is based on single‐center data, and tends to have selection biases. The data were extracted from medical records; factors such as extranodal extension of metastatic lymph nodes were not available. Furthermore, different surgeons were involved in the procedure; surgeon-specific factors, such as the number of removed lymph nodes, might affect postoperative outcomes. Third, LND was not routinely performed for all PTC patients in our institution, and occult LLNM may be present. Finally, nomograms in our study were assessed only using the internal validation method. Validation of nomograms may be compromised given the diagnostic patterns in different institutions. Thus, we will conduct prospective multi-center institutional trials in subsequent studies to obtain more objective conclusions.

In conclusion, the diameter of the tumor has an impact on the rate of LLNM. We found that LLNM in PTMC patients was independently related to gender, the number of foci, location, shape, and CLNM. For macro-PTC patients, CLT, the number of foci, location, margin, CLNM, and CLNR were all independent predictors for LLNM. By using the above variables, we constructed nomograms that can not only detect high-risk patients with occult LLNM preoperatively, but also form appropriate treatment protocols for postoperative management of PTC patients with different risks.

## Data availability statement

The raw data supporting the conclusions of this article will be made available by the authors, without undue reservation.

## Ethics statement

Written informed consent was obtained from the individual(s) for the publication of any potentially identifiable images or data included in this article. The need for informed consent was waived due to the retrospective nature of this study.

## Author contributions

J-WF and L-ZH: Writing—original draft, Software, and Data curation. S-YL: Validation, Formal analysis, and Data curation. FW: Conceptualization. JY and JH: Validation and Investigation. ZQ and YJ: Writing—review and editing, Visualization, and Supervision. All authors contributed to the article and approved the submitted version.

## Acknowledgments

Lei Qin, the English language editor, was responsible for correcting language and grammar issues.

## Conflict of interest

The authors declare that the research was conducted in the absence of any commercial or financial relationships that could be construed as a potential conflict of interest.

## Publisher’s note

All claims expressed in this article are solely those of the authors and do not necessarily represent those of their affiliated organizations, or those of the publisher, the editors and the reviewers. Any product that may be evaluated in this article, or claim that may be made by its manufacturer, is not guaranteed or endorsed by the publisher.

## Supplementary material

Supplementary Figure 1 Nomogram for predicting LLNM in all PTC patients. **(A)** preoperative nomogram; **(B)** postoperative nomogram.

Supplementary Figure 2 ROC curves for the model. **(A)** AUC was 0.730 for preoperative model of predicting LLNM in all PTC patients; **(B)** AUC was 0.758 for postoperative model of predicting LLNM in all PTC patients.

Supplementary Figure 3 Calibration curves of nomograms for predicting LLNM. **(A)** calibration curve for preoperative model of predicting LLNM in all PTC patients; **(B)** calibration curve for postoperative model of predicting LLNM in all PTC patients.
