# Supplementary figures and images for: Nomograms for the prediction of lateral lymph node metastasis in papillary thyroid carcinoma: Stratification by size
Source: Front Oncol. 2022 Sep 28;12:944414. doi: 10.3389/fonc.2022.944414 (PMC9554485; doi:10.3389/fonc.2022.944414)

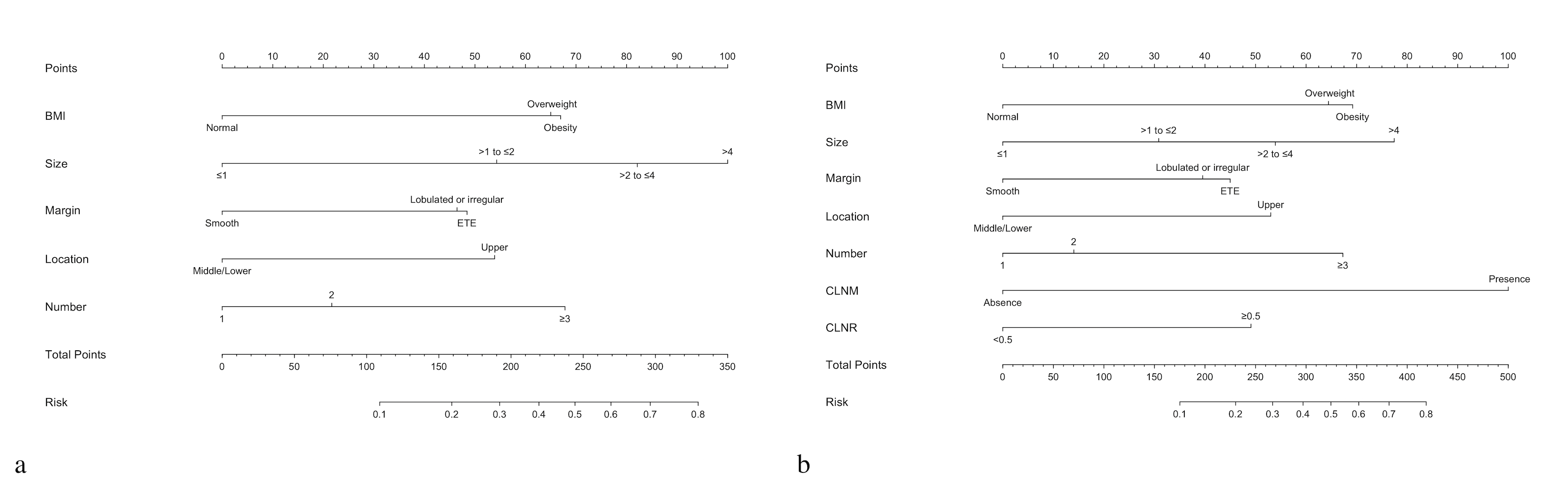

Supplement: Supplementary Figure 1 — Nomogram for predicting LLNM in all PTC patients. (A) preoperative nomogram; (B) postoperative nomogram. [file Image_1.tiff]

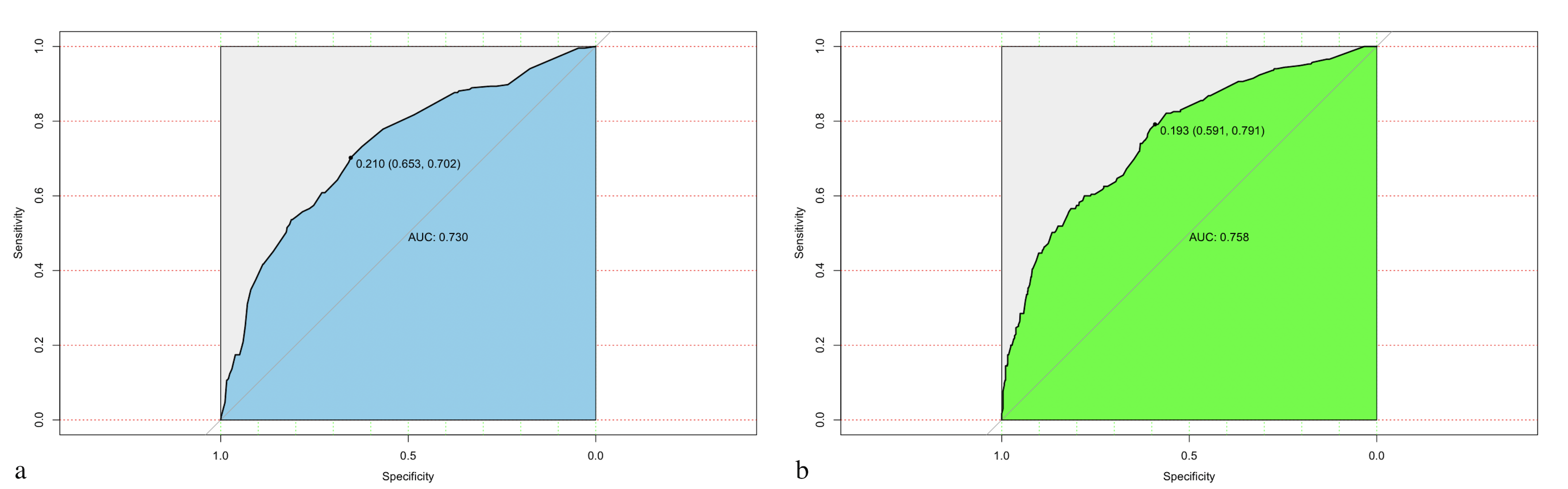

Supplement: Supplementary Figure 2 — ROC curves for the model. (A) AUC was 0.730 for preoperative model of predicting LLNM in all PTC patients; (B) AUC was 0.758 for postoperative model of predicting LLNM in all PTC patients. [file Image_2.tiff]

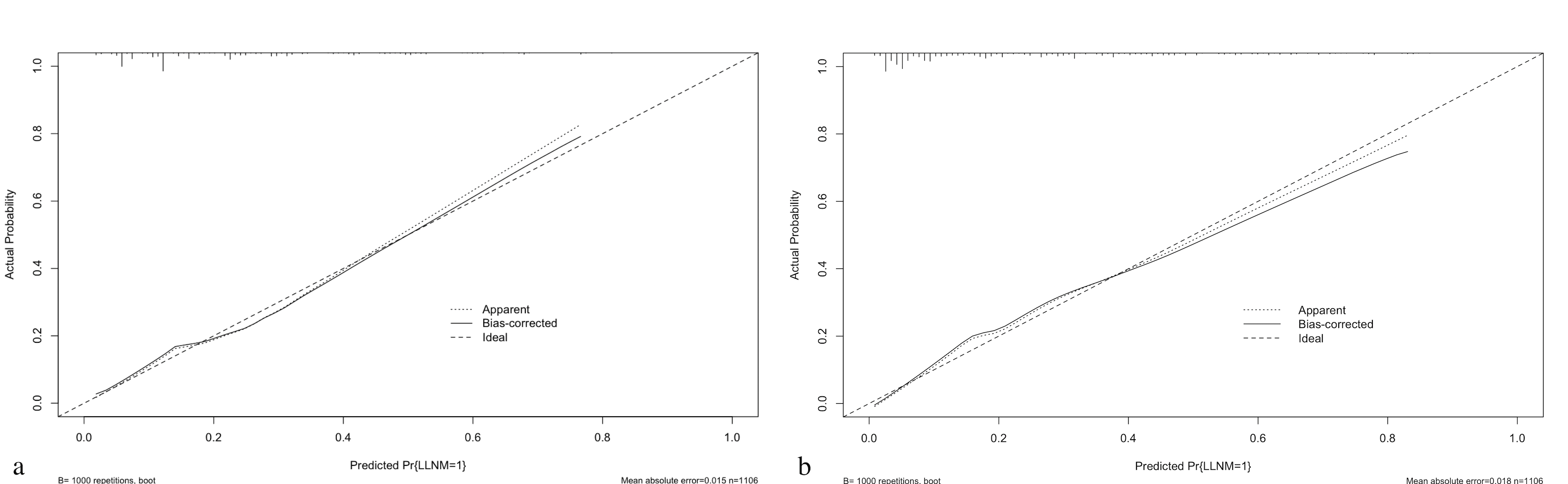

Supplement: Supplementary Figure 3 — Calibration curves of nomograms for predicting LLNM. (A) calibration curve for preoperative model of predicting LLNM in all PTC patients; (B) calibration curve for postoperative model of predicting LLNM in all PTC patients. [file Image_3.tiff]
